# Body Mass Index, Mortality, and Gender Difference in Advanced Chronic Kidney Disease

**DOI:** 10.1371/journal.pone.0126668

**Published:** 2015-05-05

**Authors:** Jiun-Chi Huang, Hugo You-Hsien Lin, Lee-Moay Lim, Szu-Chia Chen, Jer-Ming Chang, Shang-Jyh Hwang, Jer-Chia Tsai, Chi-Chih Hung, Hung-Chun Chen

**Affiliations:** 1 Department of Internal Medicine, Kaohsiung Municipal Hsiao-Kang Hospital, Kaohsiung Medical University, Kaohsiung, Taiwan; 2 Division of Nephrology, Department of Internal Medicine, Kaohsiung Medical University Hospital, Kaohsiung Medical University, Kaohsiung, Taiwan; 3 Department of Internal Medicine, Kaohsiung Municipal Ta-Tung Hospital, Kaohsiung Medical University, Kaohsiung, Taiwan; 4 Faculty of Medicine, College of Medicine, Kaohsiung Medical University, Kaohsiung, Taiwan; 5 Faculty of Renal Care, College of Medicine, Kaohsiung Medical University, Kaohsiung, Taiwan; 6 Institute of Population Sciences, National Health Research Institutes, Miaoli, Taiwan; Mayo Clinic Arizona, UNITED STATES

## Abstract

**Background and Aim:**

A higher body mass index (BMI) appears to be reversely associated with mortality in dialysis patients. Moreover, although women have better survival in chronic kidney disease (CKD), this survival advantage is cancelled in dialysis. The association between BMI and mortality and the gender difference remain controversial in advanced CKD.

**Methods:**

This study enrolled 3,320 patients (1,938 men and 1,382 women) from southern Taiwan who had CKD stages 3–5 with a BMI of 15.0–35.0 kg/m^2^.

**Results:**

During a median 2.9-year follow-up, there were 328 (16.9%) all-cause mortality and 319 (16.5%) cardiovascular (CV) events and death in male patients, 213 (15.4%) all-cause mortality and 224 (16.2%) CV events and death in female patients. Compared with the reference BMI of 27.6–30.0 kg/m^2^ in an adjusted Cox model, lower-BMI groups in men, BMI 15.0–20.0 kg/m^2^ and 20.1–22.5 kg/m^2^, were associated with higher risks of all-cause mortality: hazard ratios (HRs) 3.19 (95% confidence interval [CI], 1.97–5.18) and 2.01 (95% CI, 1.29–3.14), respectively. Higher-BMI group in men, BMI 30.1–35.0 kg/m^2^, was associated with a higher risk of all-cause mortality: HR 1.72 (95% CI, 1.02–2.96). Likewise, lower- and higher-BMI groups in men were associated with a higher risk of CV events and death. In women, these associations between BMI and poor outcomes were not observed.

**Conclusions:**

In advanced CKD, there was a reverse J-shaped association between BMI and all-cause mortality, and a U-shaped association between BMI and CV outcomes in men. Neutral associations between BMI and poor outcomes were detected in women. Gender could modify the effect of BMI on mortality in patients with CKD.

## Introduction

Obesity and overweight have been a rapid-growing and pandemic problem worldwide over the past few decades [1]. Excess weight is associated with increased mortality in the general population, primarily because of the increased risk of cardiovascular disease (CVD) [2]. Accumulating evidence has shown either a J- or U-shaped association between body mass index (BMI) and mortality [3–5]. The lowest risk of death is observed at a BMI of 20.0–27.5 kg/m^2^ [4,5], suggesting a survival advantage in individuals with a normal BMI. However, in certain disease groups, such as patients with heart failure [6], chronic obstructive pulmonary disease [7], and dialysis patients [8–10], the relationship between excess weight and death appears to be reversed.

In contrast to the general population and patients on dialysis, the relationship between BMI and mortality in patients with chronic kidney disease (CKD) remains contradictory. Some earlier studies suggested that a lower BMI was associated with an increased risk of death in CKD [11–13], whereas some investigations found no association between BMI and adverse outcomes in CKD [14–16]. Patients with advanced CKD are different from the general population and patients with earlier stages of CKD regarding the burden of cardiovascular (CV) events and mortality [17]. The risk nadir BMI and the association between BMI and mortality are not well understood in advanced CKD. Furthermore, men and women exhibit differences in the pathogenesis and clinical prognosis of many diseases [18]. In CKD, the female gender was associated with a slower decline in renal function and better renal and patient survival [11,19]; however, this survival advantage is cancelled in dialysis [20,21]. The gender difference in the relationship between BMI and mortality in advanced CKD remains unclear.

Therefore, the aims of our study were to investigate the association between BMI, CV events, and mortality and to explore gender differences in these associations in patients with CKD stages 3–5.

## Methods

### Ethics statement

The study protocol was approved by the institutional review board of the Kaohsiung Medical University Hospital (KMUH-IRB-20140076). Written informed consents were obtained from each patient, and all clinical investigations were conducted according to the principles expressed in the Declaration of Helsinki. The patients gave consent for the publication of the clinical details.

### Study design and participants

Integrated CKD care program Kaohsiung for delaying dialysis (ICKD) study was designed as a prospective cohort study to investigate the impact of an integrated CKD care program on clinical outcomes in patients with CKD stages 1–5. Exclusion criteria were acute kidney injury, which is defined as more than 50% decrease in the estimated glomerular filtration rate (eGFR) in 3 months, and long-term dialysis. 3,749 patients participated in the study from the nephrology out-patient departments of two hospitals located in southern Taiwan between November 11, 2002 and May 31, 2009, and were followed-up until July 31, 2010. Thirty patients with missing BMI data, and 356 patients with CKD stages 1 or 2 were excluded. The definition of CKD was by the National Kidney Foundation-Kidney Disease Outcomes Quality Initiative (K/DOQI) guidelines, and CKD stage was classified based on the participants’ baseline eGFR. Study participants were divided into six BMI categories as follows: 15.0–20.0, 20.1–22.5, 22.6–25.0, 25.1–27.5, 27.6–30.0 and 30.1–35.0 kg/m^2^, according to previous Asian BMI studies [5]. The extreme-BMI groups, which represented less than 1% of participants, including five subjects with a BMI lower than 14.9 kg/m^2^ and thirty-eight subjects with a BMI greater than 35.1 kg/m^2^, were excluded from the study. A total 3,320 subjects with CKD stages 3–5 and a BMI of 15.0–35.0 kg/m^2^ were enrolled and analyzed.

### Collection of demographic, medical and laboratory data

Baseline variables were collected at the baseline visit and included demographic features (age and gender), medical history (diabetes mellitus, hypertension, CVD, current smoking status, and cancer), examination findings [BMI and mean arterial pressure (MAP)], renal function status [eGFR, CKD stage, and urine protein-to-creatinine ratio (Upcr)]. Laboratory data obtained at the baseline visit and within 3 months before the baseline visit were averaged and analyzed [albumin, hemoglobin, total cholesterol, C-reactive protein (CRP), glycated hemoglobin (HbA1c), bicarbonate, and phosphorus]. Demographic features were baseline records, and medical history was obtained by a review of physician’s charts and interviews with patients. BMI was calculated as the weight in kilograms divided by the square of height in meters. Mean MAP was calculated by the mean of repeated measured MAP 3 months before study enrollment. Upcr was calculated as urine protein (mg) divided by urine creatinine (g) in a random spot urine sample. Biochemistry measurements were performed during the screening visit, the baseline visit, and every 3 months thereafter, as the protocol.

### Quantification of renal function

Kidney function was quantified using eGFR calculated by the four-variable equation in the Modification of Diet in Renal Disease (MDRD) Study [22]: eGFR mL/min/1.73 m^2^ = 186 × serum creatinine^−1.154^ × age^−0.203^ × 0.742 (if female) × 1.212 (if black patient). We classified our patients according to evidence of kidney damage lasting for more than 3 months into CKD stage 3, 4, and 5 based on eGFR levels (mL/min/1.73 m^2^) of 30–59, 15–29, and <15, respectively.

### Outcomes

Clinical outcomes, including all-cause mortality and CV events and death, were accessed. Survival status and cause of death were ascertained by reviewing death certificates using charts or the National Death Index. CV events were ascertained by reviewing charts and were defined as hospitalization for acute coronary syndrome (Deyo-modified Charlson score, ICD-9-CM: 410.x–412.x), acute cerebrovascular disease (430.x–438.x), congestive heart failure (428.x), and peripheral arterial occlusion disease (443.9, 441.x, 785.4, V43.4, procedure 38.48), followed by death by one of the aforementioned causes. Patients were not censored because of dialysis.

### Statistical analysis

Summary statistical results regarding baseline characteristics of all subjects and stratification according to BMI are expressed as percentages for categorical data, mean standard ± deviation for continuous variables with an approximately normal distribution, and median and interquartile range for continuous variables with a skewed distribution. Differences between groups were assessed by the Pearson chi-square test for categorical variables or by the one way ANOVA for continuous variables. Cox proportional hazards analysis was used to investigate the relationship of BMI with all-cause mortality and CV events. The proportional hazards assumption was tested by the Schoenfeld goodness-of-fit procedures, which did not show meaningful violations. Study participants with a BMI of 27.6–30.0 kg/m^2^ were used as the reference group because this group had the lowest all-cause mortality and CV events and death. All clinically relevant covariates were selected according to our previous publications and literature and continuous variables with a skewed distribution were log transformed to attain normal distribution. Adjusted covariates included age, gender, hypertension, CVD, diabetes mellitus, current smoking status, MAP, eGFR, log-transformed Upcr, albumin, hemoglobin, HbA1C, log-transformed total cholesterol, log-transformed CRP, and phosphorus [23–25]. Cox survival analyses with pre-specified subgroups were also performed by adding appropriate cross-product interaction terms including age, gender, CVD, CKD stages, Upcr, hemoglobin, albumin, and CRP. Statistical analysis was performed using the R 2.15.2 software (R Foundation for Statistical Computing, Vienna, Austria) and the SPSS, version 18.0 (SPSS Inc., Chicago, IL) for Windows.

## Results

### Baseline characteristics of study participants

A total of 3,320 non-dialyzed patients (1,938 men and 1,382 women) with CKD stages 3–5 were included in the study and analyzed. Baseline characteristics of study participants divided according to the categories of BMI are summarized in Tables 1 and 2.

**Table 1:** Comparison of baseline characteristics among male patients stratified on BMI categories.

				BMI (kg/m^2^)				
	All	15.0–20.0	20.1–22.5	22.6–25.0	25.1–27.5	27.6–30.0	30.1–35.0	
Characteristics	(n = 1938)	(n = 140)	(n = 319)	(n = 601)	(n = 482)	(n = 252)	(n = 144)	*P* (ANOVA)
**Demographic and medical history**								
Age (year)	63.5±13.4	65.3±16.2	65.1±13.7	64.1±12.7	62.7±13.0	63.2±13.2	59.3±13.5	< 0.001
Hypertension (%)	65.2	52.1	64.6	65.0	66.0	70.2	68.8	0.003
Diabetes mellitus (%)	43.7	37.9	39.8	43.1	45.2	47.2	48.6	0.008
Cardiovascular disease (%)	26.1	25.7	23.2	27.1	24.3	29.8	27.8	0.275
Current smoking status (%)	17.5	15.7	16.3	18.1	17.2	18.3	19.4	0.213
Cancer (%)	8.8	13.6	13.2	8.3	7.7	5.6	6.3	< 0.001
**Examination findings**								
BMI (kg/m^2^)	24.8±3.4	18.5±1.2	21.4±0.7	23.8±0.7	26.2±0.7	28.6±0.7	31.8±1.4	
MAP (mmHg)	100.3±13.9	94.8±12.8	98.1±13.7	99.9±13.5	101.2±13.2	103.3±15.2	103.2±14.2	< 0.001
**Renal function status**								
eGFR (ml/min/1.73 m^2^)	29.2±16.2	23.7±15.2	27.7±16.2	28.4±16.0	30.1±16.1	30.9±15.6	34.8±16.7	< 0.001
CKD stage								< 0.001
Stage 3 (%)	46.4	33.6	40.7	45.1	49.8	51.2	56.9	
Stage 4 (%)	26.9	27.8	30.1	25.8	24.7	27.4	29.9	
Stage 5 (%)	26.7	38.6	29.2	29.1	25.5	21.4	13.2	
Upcr (mg/g)	863 (292–2080)	972 (404–2188)	937 (299–2116)	892 (287–2375)	886 (329–1942)	728 (227–1829)	690 (230–1792)	0.011
**Laboratory data**								
Albumin (g/dL)	3.8±0.6	3.7±0.6	3.8±0.6	3.8±0.6	3.9±0.5	3.9±0.5	3.9±0.6	0.002
Hemoglobin (g/dL)	11.7±2.4	10.3±2.0	11.0±2.2	11.7±2.4	12.0±2.5	12.3±2.4	12.9±2.3	< 0.001
Total cholesterol (mg/dL)	185 (157–213)	175 (141–201)	181 (155–213)	190 (162–215)	184 (158–212)	183 (156–213)	190 (164–216)	0.066
CRP (mg/L)	1.3 (0.4–5.5)	2.2 (0.5–9.1)	1.0 (0.3–5.0)	1.1 (0.4–4.5)	1.2 (0.4–5.6)	1.6 (0.4–6.7)	1.9 (0.5–6.4)	0.265
HbA1C (%)	6.5±1.6	6.3±1.6	6.4±1.6	6.4±1.6	6.7±1.6	6.5±1.4	6.9±1.6	< 0.001
Bicarbonate (mEq/L)	22.6±4.2	21.5±4.7	22.3±4.5	22.5±4.1	22.8±3.9	22.7±4.1	23.9±4.1	< 0.001
Phosphorus (mg/dL)	4.2±1.2	4.4±1.4	4.3±1.4	4.1±1.2	4.1±1.1	4.0±1.1	4.0±1.0	< 0.001
**Outcomes**								
All-cause mortality (%)	16.9	32.1	21.3	15.0	15.1	11.5	16.0	0.002
CV events and death (%)	16.5	18.6	18.8	15.3	17.6	13.1	16.0	0.095

Data expressed as mean ± standard deviation, median (interquartile range) or percentage.

Abbreviations: BMI, body mass index; MAP, mean arterial pressure; CKD, chronic kidney disease; eGFR, estimated glomerular filtration rate; Upcr, urine protein-to-creatinine ratio; CRP, C-reactive protein; HbA1C, glycated hemoglobin; CV, cardiovascular.

**Table 2:** Comparison of baseline characteristics among female patients stratified on BMI categories.

				BMI (kg/m^2^)				
	All	15.0–20.0	20.1–22.5	22.6–25.0	25.1–27.5	27.6–30.0	30.1–35.0	
Characteristics	(n = 1382)	(n = 188)	(n = 294)	(n = 357)	(n = 261)	(n = 163)	(n = 119)	*P* (ANOVA)
**Demographic and medical history**								
Age (year)	63.3±13.7	60.2±16.5	62.1±13.6	64.5±13.3	64.7±13.4	63.8±11.8	63.5±12.3	0.015
Hypertension (%)	69.0	62.8	63.3	70.3	76.2	69.3	73.1	0.003
Diabetes mellitus (%)	44.9	34.0	37.1	47.1	47.9	54.0	56.3	< 0.001
Cardiovascular disease (%)	26.1	26.1	21.1	24.9	29.1	28.2	32.8	0.026
Current smoking status (%)	2.3	1.6	2.4	2.8	1.9	1.8	3.4	0.479
Cancer (%)	9.1	10.1	8.8	11.2	10.3	5.5	4.2	0.065
**Examination findings**								
BMI (kg/m^2^)	24.3±3.9	18.5±1.1	21.3±0.7	23.8±0.7	26.2±0.7	28.7±0.8	31.7±1.4	
MAP (mmHg)	99.5±13.4	96.1±14.2	98.8±13.6	100.5±13.5	99.4±13.0	101.1±11.4	101.3±13.5	< 0.001
**Renal function status**								
eGFR (ml/min/1.73 m^2^)	20.2±13.6	18.2±13.1	20.0±14.2	19.7±13.6	21.4±13.4	21.4±13.7	20.8±13.1	0.035
CKD stage								< 0.001
Stage 3 (%)	22.6	20.7	24.5	20.7	23.0	25.8	21.0	
Stage 4 (%)	31.8	26.1	26.9	31.7	38.7	32.5	37.8	
Stage 5 (%)	45.6	53.2	48.6	47.6	38.3	41.7	41.2	
Upcr (mg/g)	1457 (676–3207)	1549 (708–3043)	1445 (720–3208)	1513 (697–3017)	1297 (645–3050)	1449 (575–3548)	1362 (544–4565)	0.676
**Laboratory data**								
Albumin (g/dL)	3.8±0.5	3.7±0.5	3.8±0.5	3.8±0.5	3.8±0.6	3.8±0.5	3.8±0.5	0.825
Hemoglobin (g/dL)	9.9±1.8	9.4±1.7	9.7±1.8	9.8±1.7	10.2±1.8	10.3±2.0	10.5±1.9	< 0.001
Total cholesterol (mg/dL)	199 (170–232)	194 (166–221)	198 (165–230)	196 (165–229)	206 (182–242)	199 (172–235)	198 (173–232)	0.008
CRP (mg/L)	1.0 (0.4–5.1)	0.7 (0.3–3.7)	0.9 (0.3–3.4)	0.9 (0.4–4.3)	1.1 (0.4–6.0)	1.5 (0.5–7.5)	1.9 (0.5–11.3)	< 0.001
HbA1C (%)	6.4±1.6	6.0±1.4	6.2±1.5	6.3±1.5	6.7±1.9	6.7±1.5	6.9±1.6	< 0.001
Bicarbonate (mEq/L)	20.6±4.5	20.0±5.2	20.4±4.6	20.5±4.4	21.0±4.0	21.1±4.6	21.1±4.0	0.005
Phosphorus (mg/dL)	4.8±1.3	4.9±1.4	4.9±1.2	4.8±1.2	4.8±1.3	4.7±1.2	4.9±1.2	0.572
**Outcomes**								
All-cause mortality (%)	15.4	15.4	16.0	16.2	15.3	16.6	10.1	0.168
CV events and death (%)	16.2	14.9	16.7	15.7	13.0	16.6	25.2	0.079

Data expressed as mean ± standard deviation, median (interquartile range) or percentage.

Abbreviations are the same as in Table 1.

In male patients, the mean age was 63.5 ± 13.4 years, 43.7% had a history of diabetes, and 26.7% had CKD stage 5. The overall mean BMI and eGFR were 24.8 ± 3.4 kg/m^2^ and 29.2 ± 16.2 mL/min/1.73 m^2^, respectively. Compared with those with a lower BMI, male patients with a higher BMI were more likely to have a younger age, a higher prevalence of diabetes and hypertension, as well as higher levels of MAP, eGFR, albumin, hemoglobin, and HbA1C.

In female patients, the mean age was 63.3 ± 13.7 years, 44.9% had a history of diabetes, and 45.6% had CKD stage 5. The overall mean BMI and eGFR were 24.3 ± 3.9 kg/m^2^ and 20.2 ± 13.6 mL/min/1.73 m^2^, respectively. Compared with those with a lower BMI, female patients with a higher BMI were tend to have an older age, a higher prevalence of diabetes, hypertension and CVD, higher levels of MAP, eGFR, hemoglobin, CRP, and HbA1C.

### Associations between BMI and all-cause mortality

**Fig 1:**
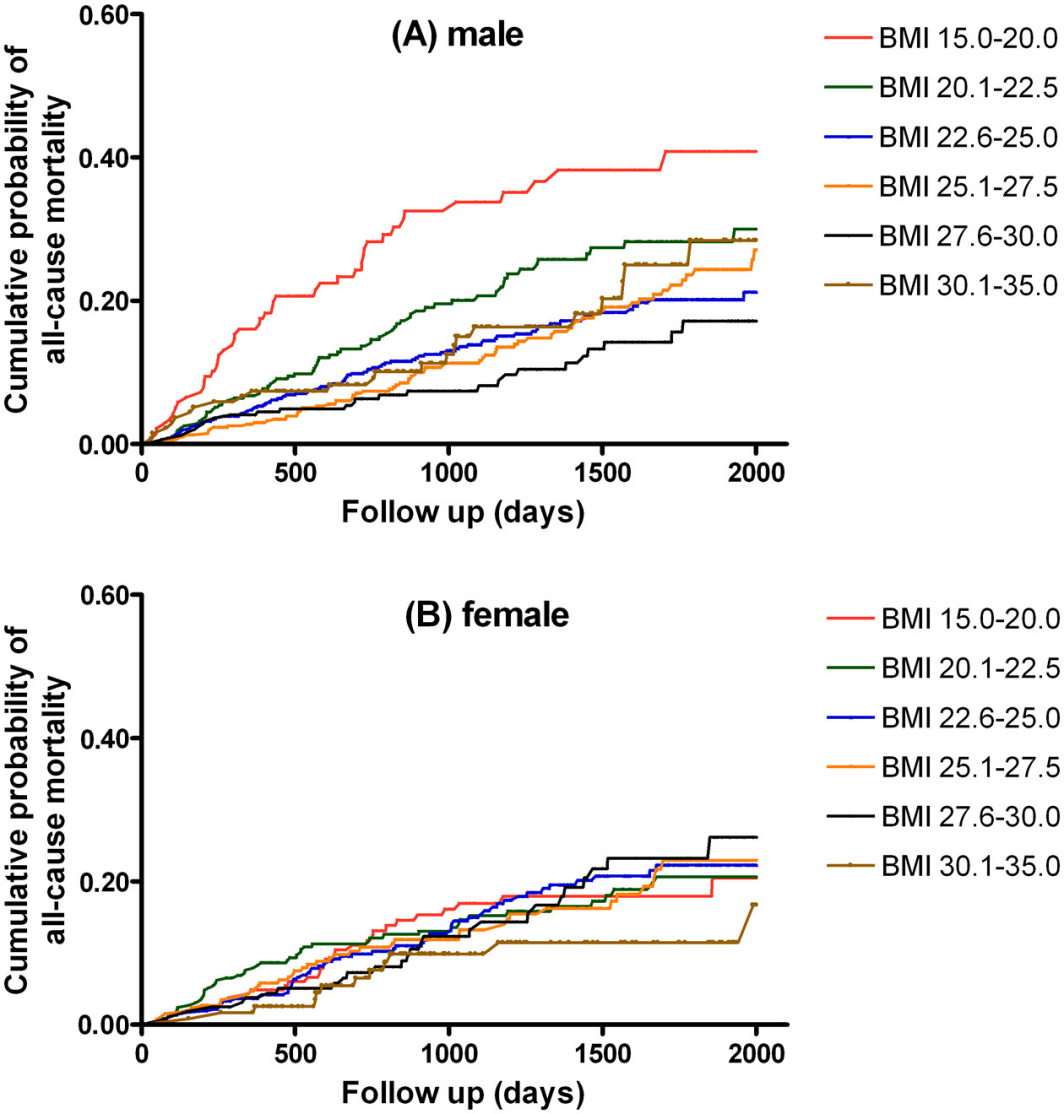
The cumulative probability of all-cause mortality using the Kaplan-Meier method in male (A) and female (B) patients according to the categories of body mass index (BMI).

There were 328 deaths (16.9%) during a median follow-up period of 2.9 (1.8–4.7) years in male patients, and 213 deaths (15.4%) during the follow-up period in female patients. We generated Kaplan-Meier curves to illustrate cumulative probability of all-cause mortality. It demonstrated that male patients with a low BMI of 15.0–20.0 kg/m^2^ had the highest cumulative probability of all-cause mortality. But such phenomenon was not observed in female patients (Fig 1).

**Fig 2:**
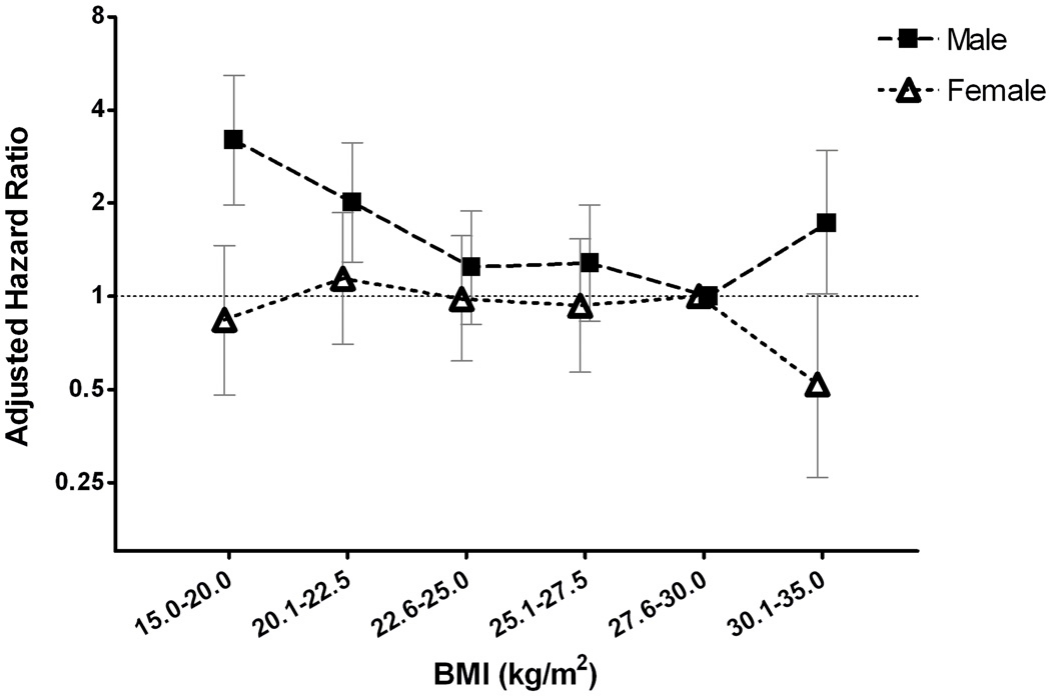
Multivariate-adjusted hazard ratios (HRs) of all-cause mortality according to the categories of body mass index (BMI) in male (■) and female (△) patients. Error bars indicate 95% confidence intervals. Multivariate-adjusted HRs were adjusted for age, gender, cardiovascular disease, diabetes mellitus, mean arterial pressure, glycated hemoglobin, log-transformed total cholesterol, current smoking status, log-transformed C-reactive protein, estimated glomerular filtration rate, log-transformed urine protein-to-creatinine ratio, albumin, hemoglobin, and phosphorus, using participants with a BMI of 27.6–30.0 kg/m^2^ as the reference group.

Table 3 shows the results of Cox proportional hazards regression analysis for all-cause mortality, CV events and death in male patients. The low-BMI groups, i.e., male patients with a BMI of 15.0–20.0 and 20.1–22.5 kg/m^2^, had significantly increased risks of all-cause mortality, with a hazard ratio (HR) (95% confidence interval [CI]) of 3.19 (1.97–5.18), *P* < 0.001; and 2.01 (1.29–3.14), *P* = 0.002, respectively, compared with a BMI of 27.6–30.0 kg/m^2^ in the fully adjusted model. The high-BMI group (BMI of 30.1–35.0 kg/m^2^) also had an increased risk of all-cause mortality, with a HR (95% CI) of 1.72 (1.02–2.96), *P* = 0.042, compared with a BMI of 27.6–30.0 kg/m^2^ in the fully adjusted model. It showed a reverse J-shaped association between BMI and all-cause mortality in male patients (Fig 2).

**Table 3:** Association between BMI and clinical outcomes in male patients.

	BMI (kg/m^2^)					
	15.0–20.0	20.1–22.5	22.6–25.0	25.1–27.5	27.6–30.0	30.1–35.0
**All-cause mortality**						
Unadjusted HR (95% CI)	3.54 (2.22–5.65)^†^	2.08 (1.35–3.21)^†^	1.34 (0.88–2.04)	1.35 (0.88–2.07)	1 (Reference)	1.54 (0.89–2.66)
Adjusted HR (95% CI)	3.19 (1.97–5.18)^†^	2.01 (1.29–3.14)*	1.24 (0.81–1.89)	1.28 (0.83–1.97)	1 (Reference)	1.72 (1.02–2.96)*
**CV events and death**						
Unadjusted HR (95% CI)	2.39 (1.63–3.51)^†^	1.65 (1.17–2.33)*	1.20 (0.87–1.65)	1.29 (0.92–1.79)	1 (Reference)	1.38 (0.90–2.12)
Adjusted HR (95% CI)	1.95 (1.31–2.90)^†^	1.61 (1.13–2.29)*	1.07 (0.77–1.48)	1.25 (0.89–1.74)	1 (Reference)	1.54 (1.00–2.37)*

Values expressed as hazard ratio (HR) and 95% confidence interval (CI).

Full-adjusted model: adjusted for age, hypertension, cardiovascular disease, diabetes mellitus, MAP, HbA1C, log-transformed total cholesterol, current smoker, log-transformed CRP, eGFR, log-transformed Upcr, albumin, hemoglobin and phosphorus.

Abbreviations are the same as in Table 1.

*P < 0.05 compared with reference BMI category.

^†^ *P* < 0.001 compared with reference BMI category.

Compared with those with a BMI of 27.6–30.0 kg/m^2^, female patients with a lower BMI did not show significantly increased risk of all-cause mortality in the fully-adjusted model. However, the high-BMI group (BMI of 30.1–35.0 kg/m^2^) exhibited a trend of decreased risk of all-cause mortality, with a HR (95% CI) of 0.52 (0.26–1.02), *P* = 0.057 (Table 4). The relationship between BMI and all-cause mortality in female patients showed a nearly flat curve (Fig 2)

**Table 4:** Association between BMI and clinical outcomes in female patients.

	BMI (kg/m^2^)					
	15.0–20.0	20.1–22.5	22.6–25.0	25.1–27.5	27.6–30.0	30.1–35.0
**All-cause mortality**						
Unadjusted HR (95% CI)	0.95 (0.57–1.61)	0.97 (0.61–1.56)	1.00 (0.63–1.58)	0.96 (0.59–1.57)	1 (Reference)	0.62 (0.31–1.22)
Adjusted HR (95% CI)	0.84 (0.48–1.46)	1.14 (0.70–1.86)	0.98 (0.62–1.57)	0.93 (0.57–1.53)	1 (Reference)	0.52 (0.26–1.02)
**CV events and death**						
Unadjusted HR (95% CI)	0.89 (0.59–1.34)	0.94 (0.65–1.35)	0.94 (0.66–1.34)	0.84 (0.57–1.24)	1 (Reference)	1.15 (0.74–1.79)
Adjusted HR (95% CI)	0.78 (0.51–1.20)	1.08 (0.74–1.58)	0.97 (0.68–1.40)	0.79 (0.53–1.17)	1 (Reference)	1.03 (0.66–1.60)

Values expressed as hazard ratio (HR) and 95% confidence interval (CI).

Full-adjusted model: adjusted for age, hypertension, cardiovascular disease, diabetes mellitus, MAP, HbA1C, log-transformed total cholesterol, current smoker, log-transformed CRP, eGFR, log-transformed Upcr, albumin, hemoglobin and phosphorus.

Abbreviations are the same as in Table 1.

*P < 0.05 compared with reference BMI category.

^†^ *P* < 0.001 compared with reference BMI category.

### Associations between BMI and CV outcomes

**Fig 3:**
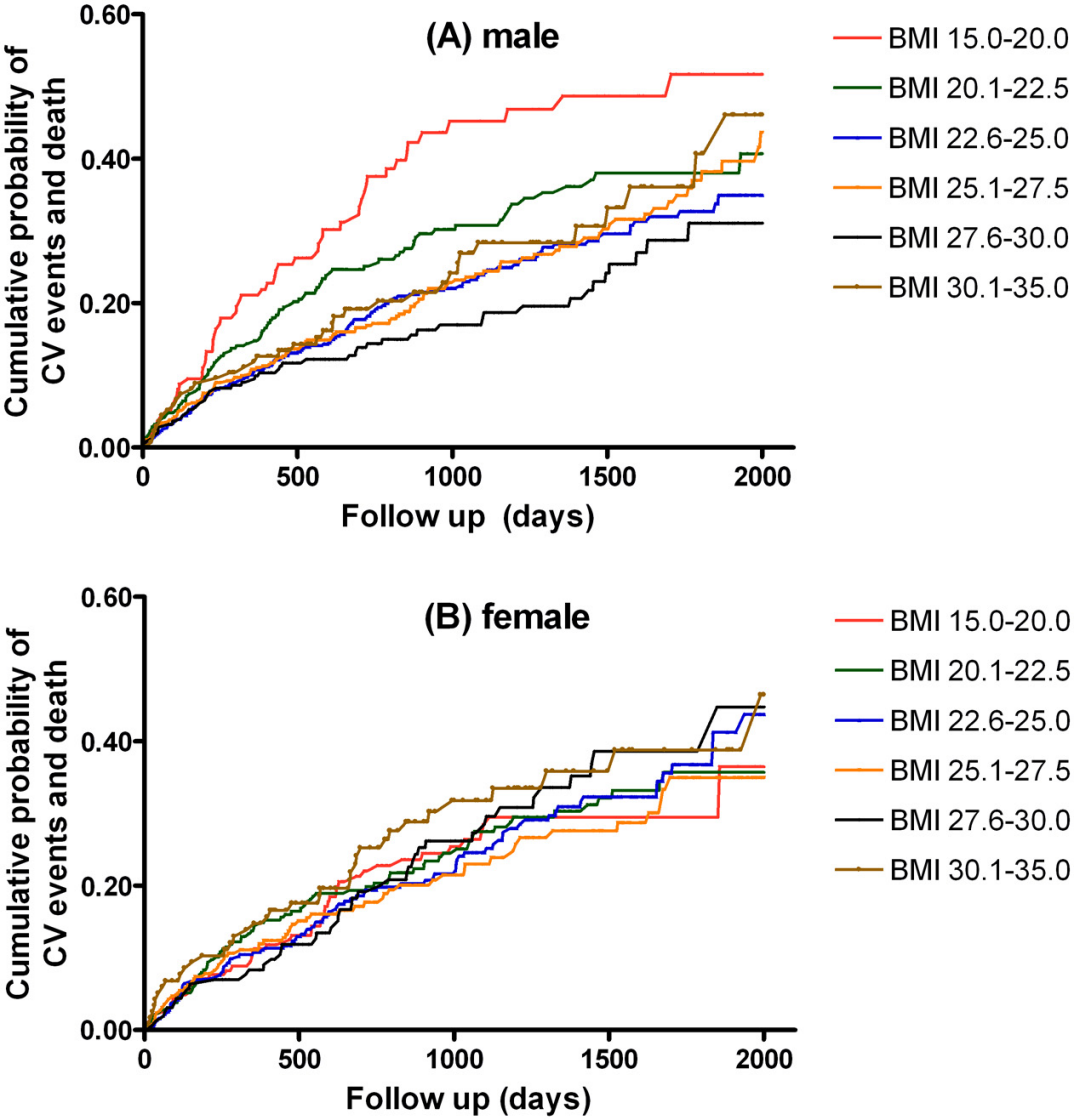
The cumulative probability of cardiovascular (CV) events and death using the Kaplan-Meier method in male (A) and female (B) patients according to the categories of body mass index (BMI).

During the follow-up period, there were 319 CV events and death (16.5%) in male patients, and 224 CV events and death (16.2%) in female patients. The Kaplan-Meier curves illustrating cumulative probability of CV events and death showed that male patients with a low BMI of 15.0–20.0 kg/m^2^ had the highest cumulative probability of poor CV outcomes. But this relationship was not found in female patients (Fig 3).

**Fig 4:**
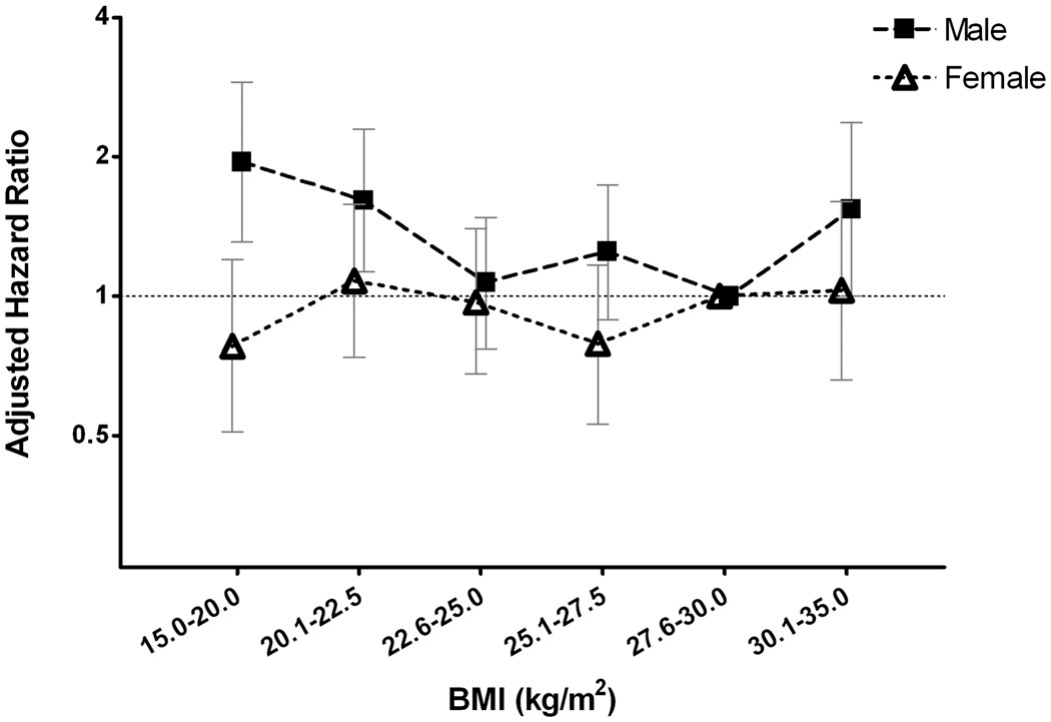
Multivariate-adjusted hazard ratios (HRs) of cardiovascular (CV) events and death according to the categories of body mass index (BMI) in male (■) and female (△) patients. Error bars indicate 95% confidence intervals. Multivariate-adjusted HRs were adjusted for age, gender, cardiovascular disease, diabetes mellitus, mean arterial pressure, glycated hemoglobin, log-transformed total cholesterol, current smoking status, log-transformed C-reactive protein, estimated glomerular filtration rate, log-transformed urine protein-to-creatinine ratio, albumin, hemoglobin, and phosphorus, using participants with a BMI of 27.6–30.0 kg/m^2^ as the reference group.

The low-BMI groups in male patients with a BMI of 15.0–20.0 and 20.1–22.5 kg/m^2^, had significantly increased risks of CV events and death, with a hazard ratio (HR) (95% CI) of 1.95 (1.31–2.90), *P* < 0.001; and 1.61 (1.13–2.29), *P* = 0.008, respectively, compared with a BMI of 27.6–30.0 kg/m^2^ in the fully adjusted model. The high-BMI group in male patients (BMI of 30.1–35.0 kg/m^2^) also demonstrated an increased risk of CV events and death, with a HR (95% CI) of 1.54(1.00–2.37), *P* = 0.049, compared with a BMI of 27.6–30.0 kg/m^2^ in the fully adjusted model (Table 3). The multivariate-adjusted association between BMI and CV outcomes in male patients was U-shaped (Fig 4).

Compared with those with a BMI of 27.6–30.0 kg/m^2^, female patients with a lower or a higher BMI did not show significantly increased risk of CV events and death in the fully-adjusted model (Table 4). The association between BMI and CV outcomes in female patients showed a nearly flat curve (Fig 4).

## Discussion

In the present study, we investigated the relationship of BMI with mortality, CV events and death in Asian patients with CKD stages 3–5. We found gender differences in associations between BMI, all-cause mortality, and CV events and death. There was a reverse J-shaped association between BMI and all-cause mortality, and a U-shaped association between BMI and CV outcomes in male patients with advanced CKD. By contrast, the associations of BMI with all-cause mortality and CV outcomes in female patients were illustrated as nearly flat curves.

Although emerging evidence indicates a survival advantage for a high BMI in dialysis patients, the role of BMI in CKD patients and gender differences has not been described well. Kovesdy et al. [12] showed that a lower BMI (<22.2 kg/m^2^) was associated with mortality in 521 veterans mainly with CKD stage 3 or 4. Furthermore, Kwan et al. [13] found an association between increased BMI and lower mortality among 461 participants, the majority of whom had CKD stage 3, in the Atherosclerosis Risk in Communities (ARIC) study. An inverse relationship between excess weight and mortality appears to be evident in CKD. However, some studies did not support this paradoxical association [14,26]. These discrepancies may be explained by the effects of comorbid conditions, racial differences, and severity of CKD among the study cohorts. Despite a limited number of studies in advanced CKD, Evans et al. [11] reported an inverse relationship between BMI and mortality in patients with CKD stages 4–5. Our findings in male patients with advanced CKD were partly comparable with those reported by Kovesdy et al. and Evans et al., suggesting a low BMI increased mortality risk.

In the present study, we found a U-shaped association between BMI and CV outcomes in male patients, whereas previous studies of Caucasian cohorts showed no association between BMI and CV outcomes [14,15]. The reasons for these different associations are not clear but may be related to racial disparities. Investigations based on the US Renal Data System data showed that Asian-American ESRD patients do not have better survival at a high BMI and reported a U-shaped association in this population [27,28]. Obesity may exert race-specific effects on survival advantage in CKD and dialysis patients. The associations between BMI and health risks may differ between Asian and European populations [29]. Moreover, it has been shown that Asians develop a higher rate of diabetes than do white populations at a BMI of 30 kg/m^2^ [30]. Given the impact of obesity and diabetes on CV risk, a stronger association between a high BMI and CVD in Asians compared with the Western population is expected, as in the present study.

In contrast to reverse J-shaped or U-shaped associations between BMI and poor outcomes in men, female patients displayed overall neutral associations. Sex hormone may contribute to the different severity of inflammation and wasting among men and women. A low BMI usually reflects malnutrition and inflammation, leading to endothelial dysfunction in pre-dialysis and dialysis patients [31,32]. Estrogen has anti-inflammatory and immunomodulatory effects, as it modulates proinflammatory cytokines, chemokines, and adhesion molecules and reduces oxidative stress [33–35]. Testosterone deficiency, which has been associated with muscle wasting and inflammation in men with CKD [36], may also play an important role. Previous reports showed that reduced muscle mass and elevated CRP were associated with a lower survival rate in male, but not in female CKD patients close to the start of dialysis [37]. Loss of muscle mass in conjunction with presence of inflammation might be a much more detrimental sign in men. These hormonal and metabolic derangements might account for the positive association between low BMI with poor outcomes in men, but not in women with CKD.

Furthermore, gender differences in body composition might be another possible explanation, as women have a higher percentage of body fat compared with men at an equivalent BMI [38]. A higher fat mass was associated with a lower risk of mortality in dialysis patients [39,40]. The favorable effects of increased fat mass were presumably due to higher metabolic reserves and sequestration of uremic toxins, thus providing energy that is required for survival in ill conditions, such as those with advanced CKD and dialysis patients. Taken together, it appears to be an interaction between sex hormones and metabolic factors, regarding the gender differences in associations between BMI and poor outcomes in CKD.

There were several limitations in the present study. First, we used the baseline BMI for analysis. Data of time-dependent changes in BMI were not obtained, and we were not able to investigate the association between weight variation and outcomes. Second, the study subjects had advanced CKD; thus, the results may not be generalizable to all CKD populations. Third, although premature death is often observed in CKD, the observation period was relatively short in the present study. Third, BMI is limited to differentiate the fat and lean mass. Therefore, body composition analysis using dual-energy X-ray absorption is of importance to elucidate in the future studies. Moreover, BMI may be misleading in the presence of edema, which occurs commonly in patients with advanced CKD. Waist-to-hip ratio or conicity index may be a more sensitive marker for risk stratification in CKD [41,42].

In conclusion, our findings suggest a reverse J-shaped association between BMI and all-cause mortality, and a U-shaped association between BMI and CV outcomes in male patients with advanced CKD. However, neutral associations were detected in female patients. Hormonal and metabolic derangements might play a role in gender differences in associations between BMI and poor outcomes in CKD.
